# Chromosome Genome Sequencing and Comparative Transcriptome-Based Analyses of *Kloeckera apiculata* 34-9 Unveil the Potential Biocontrol Mechanisms Against Citrus Green Mold

**DOI:** 10.3389/fmicb.2021.752529

**Published:** 2021-11-09

**Authors:** Zhonghuan Tian, Yujie Du, Fan Yang, Juan Zhao, Shuqi Liu, Deyao Zhang, Chao-an Long

**Affiliations:** ^1^Key Laboratory of Horticultural Plant Biology of Ministry of Education, Huazhong Agricultural University, Wuhan, China; ^2^National R&D Center for Citrus Preservation, Huazhong Agricultural University, Wuhan, China; ^3^National Centre of Citrus Breeding, Huazhong Agricultural University, Wuhan, China

**Keywords:** *Kloeckera apiculata*, genome, transcriptome, biocontrol, stress

## Abstract

Biological control is an environmentally friendly, safe, and replaceable strategy for disease management. Genome sequences of a certain biocontrol agent could lay a solid foundation for the research of molecular biology, and the more refined the reference genome, the more information it provides. In the present study, a higher resolution genome of *Kloeckera apiculata* 34-9 was assembled using high-throughput chromosome conformation capture (Hi-C) technology. A total of 8.07 M sequences of *K. apiculata* 34-9 genome was anchored onto 7 pesudochromosomes, which accounting for about 99.51% of the whole assembled sequences, and 4,014 protein-coding genes were annotated. Meanwhile, the detailed gene expression changes of *K. apiculata* 34-9 were obtained under low temperature and co-incubation with *Penicillium digitatum* treatments, respectively. Totally 254 differentially expressed genes (DEGs) were detected with low temperature treatment, of which 184 and 70 genes were upregulated and downregulated, respectively. Some candidate genes were significantly enriched in ribosome biosynthesis in eukaryotes and ABC transporters. The expression of gene *Kap003732* and *Kap001595* remained upregulated and downregulated through the entire time-points, respectively, indicating that they might be core genes for positive and negative response to low temperature stress. When co-incubation with *P. digitatum*, a total of 2,364 DEGs were found, and there were 1,247 upregulated and 1,117 downregulated genes, respectively. Biosynthesis of lysine and arginine, and phenylalanine metabolism were the highest enrichment of the cluster and KEGG analyses of the co-DEGs, the results showed that they might be involved in the positive regulation of *K. apiculata* 34-9 response to *P. digitatum*. The completeness of *K. apiculata* 34-9 genome and the transcriptome data presented here are essential for providing a high-quality genomic resource and it might serve as valuable molecular properties for further studies on yeast genome, expression pattern of biocontrol system, and postharvest citrus storage and preservation.

## Introduction

*Hanseniaspora* species, including *H. uvarum*, *H. vineae*, and *H. guilliermondii*, are widely distributed in various environments with lemon-shaped cell morphology, and they are broadly known to be plentiful in wine fermentation and have a great influence on wine quality compared to other non-*Saccharomyces* yeast species (Langenberg et al., 2017; Guaragnella et al., 2020). Their abilities to provide enhanced levels of acetate esters, benzenoids, and other valuable compounds make them very promising fermentation strains (Tristezza et al., 2016; Martin et al., 2018). Moreover, enough studies had reported that *H. uvarum* (anamorph *Kloeckera apiculata*), one of the extensively known apiculata yeasts, could be frequently detected on mature fruits (especially on grapes), fermented beverages, soils, and plants (Valles et al., 2007; Zott et al., 2008). In addition, *H. uvarum* exhibited strong antagonistic properties against some phytopathogens which were responsible for citrus, strawberries, grapes, and plum fruit spoilage, thus, it was regarded as a potential biological control agent (Long et al., 2005; Cai et al., 2015; Apaliya et al., 2017; Zhang et al., 2017). In our previous studies, *K. apiculata* 34-9 (CCTCC M204025) showed biocontrol efficacy against *Penicillium digitatum*, *P. italicum*, and *Botrytis cinerea* (Long et al., 2005; Liu P. et al., 2014), and its biological control mechanisms had been studied including secretion of antifungal compounds (Liu P. et al., 2014), induction of citrus fruit resistance (Liu P. et al., 2016), and competition for nutrients with the fungi (Liu P. et al., 2013). Genome sequences of *K. apiculata* 34-9 were obtained using the Illumina HiSeq 2000 platform, a total of 8.1 M sequences were assembled into 41 scaffolds, and 3,786 protein-coding genes were defined after genome assembly and annotation (Chen et al., 2017). However, knowledge of the genome version at the chromosomal level is lacking, and the completeness of the genome is essential for providing high-quality genomic resources for further studies.

Abiotic and biotic stresses are the threatening environmental restrictions of all organisms (Suzuki et al., 2014; Bai et al., 2018; Sies and Jones, 2020; Roeber et al., 2021). They also play important roles in the inhibitory effect of antimicrobial agents. Heat stress treatment adversely affects biocontrol efficiency of *Debaryomyces hansenii* against kiwifruit gray mold, and it showed decreased expression of genes related to stimulus response and developmental process (Dai et al., 2021). The transcriptome profiles of *S. cerevisiae* and *S. kudriavzevii* showed diverse responses to cold shock of these two yeast strains, the latter strain exhibited increased translation rate in cold adaptation (Tronchoni et al., 2014). Low temperature may influence the energy absorption and cause metabolic imbalance in yeast cells, and it could decrease their biocontrol efficiency against phytopathogens. So, the relevant tolerance mechanisms of yeast exposed to low temperature are important for the food industry. However, the elucidation of cold resistance mechanisms of *K. apiculata* and the genes involved were still largely unknown.

Genome analyses have been conducted to study the genetics and molecular biology of different *Hanseniaspora* species recently. High similarity in size and genes annotation were revealed within several strains of *H. vineae*, and that it diverged from the *Saccharomyces* complex clade before the whole-genome duplication event (Giorello et al., 2019). Seixas et al. (2019) found that *H. guiliermondii* was destitute of the functional gluconeogenesis or glyoxylate cycle, and it was believed to be utilized in wine-making and other relevant biotechnological industrial processes. Accessibility of these sequenced genomic resources could therefore contribute to a better comprehension of these species on molecular and genetic aspects. This would affect genetic modification on fermentation traits and facilitate the application of these yeasts.

Here we established a chromosome-level assembly of the genome of *K. apiculata*, using a combination of next-generation sequencing and Hi-C (high-throughput chromosome conformation capture) scaffolding, which had been successfully utilized for the assembly of plant, animal, and fungal genomes (Dekker et al., 2013; Shao et al., 2018; Peng et al., 2019; Yang et al., 2020), to study key questions about *K. apiculata* genome biology that have been previously difficult to address due to the fragmentary genome assembly. Meanwhile, transcriptome analyses were performed to gain more knowledge on the growth of *K. apiculata* at low temperature, and to better understand the possible biocontrol mechanisms of *K. apiculata* against *P. digitatum*. These findings may reveal comprehensions into the molecular mechanisms of low temperature tolerance in *K. apiculata* and provide diverse transcriptome resources related to low temperature tolerance and biotic stress research.

## Materials and Methods

### Yeast Strain and Culture Conditions

The antagonist yeast *K. apiculata* 34-9 was isolated and preserved in a postharvest laboratory of Huazhong Agricultural University. It was cultured on YPDA (1% yeast extract, 2% peptone, 2% dextrose, 2% agar) plate for 2 days, or YPD medium (1% yeast extract, 2% peptone, 2% dextrose) in a rotary shaker coupled with 150 rpm shaking for 1 day at 25°C, respectively.

### Pathogen Strain and Culture Conditions

The plant pathogen *P. digitatum* N1 which was isolated and preserved in a postharvest lab of Huazhong Agricultural University was used in this study. An even mycelial layer was obtained according to a previous study (Tian et al., 2020). Briefly, 1 mL spore suspensions at a concentration of 1.0 × 10^6^ CFU/mL of *P. digitatum* was transferred into 100 mL liquid PDA medium (below 50°C) and immediately divided into Petri dishes followed by absolutely mixed. The Petri dishes were air-dried, then incubated in a climate chamber for 4 days at 25°C.

### Chromosomal Genome Assembly Using High-Throughput Chromosome Conformation Capture

The Hi-C sequencing approach was explored to obtain high-quality assembly and annotated genome. Fresh *K. apiculata* 34-9 cell samples grown on YPDA plates were harvested after incubation for 2 days at 25°C, then fixed in 1.0% formaldehyde at room temperature for crosslinking to maintain its cellular three-dimensional structure. The yeast cells were lysed, and the cross-linked DNA obtained was then digested with *Hin*dIII restriction enzyme. The digested DNA ends were filled and labeled with biotin, then ligated to form a circular chimeric molecule, and the DNA was purified and cut into 300–700 bp fragments. The density and insert size of the constructed library was determined using Qubit 2.0 (Thermo Fisher Scientific Inc., Waltham, United States) and Agilent 2100 Bioanalyzer system (Agilent Technologies Inc., Santa Clara, United States). Then the constructed library was processed for paired-end sequencing on the Illumina Hiseq X Ten platform. The Hi-C reads were filtered using Hi-C Pro to validate paired-end reads (Servant et al., 2015). In addition, the draft contigs obtained from Hi-C sequencing were grouped, ordered, and oriented for chromosomes assembly using the software Lachesis with default parameters (Burton et al., 2013).

### Identification of Repetitive Elements

Tandem repeats were identified with the utilization of a Tandem Repeat Finder (TRF) in the genome of *K. apiculata* 34-9 (Benson, 1999). Transposon elements (TEs) of *K. apiculata* 34-9 genome were annotated with the combination of homology and *de novo* approaches. Briefly, RepeatMasker^1^ and RepeatProteinMask were used for the annotation of TEs of *K. apiculata* 34-9 genome based on the RepBase database^2^ and RepeatMasker package TE protein database, respectively. RepeatModeler and LTR_FINDER were applied for *de novo* repeat prediction (Xu and Wang, 2007), and RepeatMasker was utilized for the annotation of TEs with default parameters.

### Genome Annotation

*De novo* gene prediction and homology-based strategy were employed to perform the gene prediction and functional annotation, and these results were integrated into a gene model using MAKER2 (Holt and Yandell, 2011). The completeness and accuracy of the assembly was assessed using CEGMA (Parra et al., 2007), and the final reliable gene set was obtained in a HiCESAP process. Protein sequences of *H. guilliermondii* (accession number: GCA_900119595.1), *H. opuntiae* (accession number: GCA_001749795.1), *H. uvarum* (accession number: GCA_001747055.1), *H. valbyensis* (accession number: GCA_001664025.1) genomes were aligned to the genome of *K. apiculata* 34-9 to perform homology-based gene prediction. All predicted protein-coding sequences were aligned to the SwissProt, non-redundant protein (NR), Gene Ontology (GO), InterPro, Kyoto Encyclopedia of Genes and Genomes (KEGG), and TrEMBL databases for further annotation using BLASTP with default parameters.

For annotation of non-coding RNA, tRNAscan-SE was used to screen the tRNA of *K. apiculata* 34-9 genome, and BLASTN was applied to identify rRNA based on sequence alignment with homologous species, micro-RNA (miRNA) and small nuclear RNA (snRNA) were annotated by INFERNAL from Rfam.^3^

### Comparative Genomic Collinearity Analysis

The re-assembled genome of *K. apiculata* 34-9 was compared with *H. uvarum* (assembly GCA_001747055.1) and *S. cerevisiae* (assembly GCA_002057635.1). Collinearity among these three yeasts was performed using TBtools (Chen et al., 2020) with default parameters.

### Samples Preparation for RNA-Sequencing

For low temperature (LT) treatment, yeast samples were collected after incubation for 1 day in YPD at 25°C, the cells were rapidly frozen in liquid nitrogen after centrifugation at 3,000 × g for 10 min, and marked as 0 h, then stored at −80°C. From that time, some YPD flasks were placed into a 4°C incubator for continuous incubation, and yeast samples were harvested after 5, 12, 24, and 48 h, respectively. Yeast grown at 25°C (normal temperature, Kap) was handled as the same procedure.

For the preparation of interaction between antagonist *K. apiculata* 34-9 and *P. digitatum* N1 (Pdi), yeast samples harvested after incubation for 1 day in YPD at 25°C were adjusted to a concentration of 1.0 × 10^8^ CFU/mL, and a total of 1 mL *K. apiculata* cell suspension was inoculated to each *P. digitatum* plate and continuously cultured at 25°C, from that time (marked 0 h), samples were harvested as the above time course. There were three biological replicates of each sample. All samples were rapidly frozen in liquid nitrogen and immediately stored at −80°C.

### RNA Sequencing

Total RNA from collected yeast samples was isolated using TRIzon Reagent (Cwbiotech, China) following the manufacturer’s instruction. RNA concentration and integrity were measured using NanoDrop 2000 (Thermo Fisher Scientific Inc., Waltham, United States) and Agilent 2100 Bioanalyzer system (Agilent Technologies Inc., Santa Clara, United States), respectively. RNA-sequencing (RNA-seq) libraries were prepared using a NEBNext Ultra RNA Library Prep Kit for Illumina (New England BioLabs^®^ Inc.) according to the manufacturer’s guide, and the libraries were sequenced on an Illumina HiSeq X 10 platform (Illumina Inc., San Diego, CA). The generated reads were trimmed to eliminate adaptors and enhance quality. The filtrated clean reads were mapped to *K. apiculata* 34-9 genome using Hisat2 (version 2.1.0) and aligned to the generated transcriptome assembly using Bowtie2 (version 2.2.2), respectively. The output results were estimated using RSEM (version 1.3.0) to obtain normalized counts of gene expression level. The values of expression genes were calculated as fragments per kilobase per million mapped reads (FPKM).

### Bioinformatics Analyses of Differentially Expressed Genes

The analyses of differentially expressed genes (DEGs) were conducted using R package DEseq2, and the DEGs were selected if their false discovery rate (FDR) < 0.05 and a log2 fold change > 1, then the DEGs were annotated to GO terms (Ye et al., 2018) and KEGG enrichments (Xie et al., 2011) for functional analyses.

### Validation of RNA-Sequencing Data by Quantitative Real-Time PCR

Quantitative real-time PCR (qRT-PCR) assays were conducted to confirm RNA-seq results independently. Total RNA was extracted with Fungal RNA Kit (OMEGA Biotech, Guangzhou, China) followed by the operating instructions. The first-strand cDNA synthesis was conducted using a PrimeScript^TM^ RT reagent Kit with gDNA Eraser (TaKaRa Biotechnology, Dalian, China). The PowerUP^TM^ SYBR^TM^ Green Master Mix (Life Technologies, United States) was used for the detection of the relative expression profiles according to the recommended procedures. qRT-PCR was performed on a Q6 Flex Real-Time PCR system (Life Technologies, United States). The relative gene expression level was analyzed by the 2^–ΔΔCt^ method (Livak and Schmittgen, 2001). The β*-actin* was used as a reference gene for normalization, all qRT-PCR primers were designed using Primer-BLAST^4^ and shown in Supplementary Table 1.

### Data Availability

The Hi-C sequencing and RNA-seq data generated in this study were released in NCBI Sequence Read Archive database^5^ under accession numbers PRJNA667993 and PRJNA668195.

## Results

### Genome Assembly and Annotation

**FIGURE 1:**
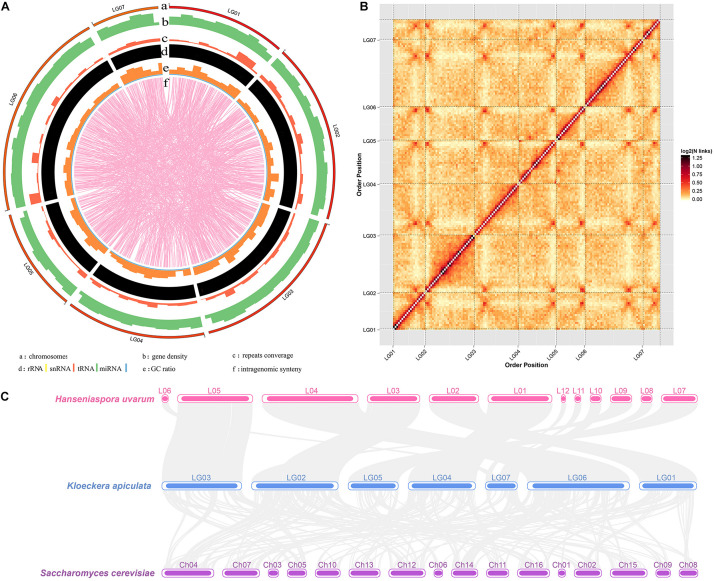
Chromosome resolution genome of *K. apiculata*. Genome synteny of *Hanseniaspora uvarum*, *K. apiculate*, and *Saccharomyces cerevisiae* are obtained from scaffolds of *H. uvarum*, and chromosomes of *K. apiculata* 34-9 and *S. cerevisiae*.

The next-generation sequencing (previous data) and Hi-C scaffolding were combined to improve *K. apiculata* yeast genome assembly and chromosome anchoring accuracy. A total of 3.47 Gb (428.40 × coverage) clean data were generated after quality control. *K. apiculata* genome was assembled into 8.1 Mb of scaffolds sequences and the assembly contained 15 scaffolds (more than 1 kb per scaffold), with a scaffold N50 of 1,349,021 bp (about 4.8-fold larger than that of the previous version), and the maximum scaffold length was 1,727,301 bp (Table 1). Meanwhile, the assembly comprised 155 contigs (more than 1 kb per contig), with a contig N50 of 104,210 bp, and the maximum contig length was 553,666 bp. The GC content was 31.98%, and there were 4,014 protein-coding genes of the new genome version, which was 228 more than the previous annotation version. In addition, 99.51% (8.06 Mb) of the genome sequences had been divided into seven groups (Supplementary Table 2). As many as 75.55% of the reads were uniquely mapped to the genome of *K. apiculata* 34-9, which were good enough for further analyses. A Hi-C contact heatmap was visualized to confirm the accuracy of Hi-C assembly at 20 kb resolution, all bins could be clearly anchored onto seven pseudochromosomes, the intensity of red color represents the number of Hi-C links on the pseudochromosomes of final assembly (Figures 1A,B). In addition, synteny blocks among *K. apiculata* 34-9, *H uvarum* and *S. cerevisiae* were visualized (Figure 1C).

**TABLE 1:** Statistics of the *Kloeckera apiculata* draft genome assembly assisted by Hi-C sequencing.

**Features**	**This version**	**Previous version**
Number of assembled contigs	155	106
Assembled contigs	8,082,270	8,093,879
Largest contig (bp)	553,666	1,362,551
Contig N50 (bp)	104,210	140,302
Contig N90 (bp)	26,703	
Number of assembled scaffolds	15	41
Assembled scaffolds	8,100,006	8,099,786
Largest scaffold (bp)	1,727,301	
Scaffold N50 (bp)	1,349,021	279,578
Scaffold N90 (bp)	850,161	
Total gap length (bp)	17,736	
Repetitive sequences (%)	1.65	0.37
GC content (%)	31.98	31.95
Genes	4,014	3,786
Average gene length (bp)	1530.63	1,627
Chromosomes	7	

*Number of assembled contigs of This version and Previous version were calculated using sequences > 1 kb and 500 bp, respectively. Number of assembled contigs, Assembled contigs, Number of assembled scaffolds, Assembled scaffolds, N50 and N90 values of the genome assembly (this version) were calculated using sequences > 1 kb.*

Repeat elements are important parts of the genome. A total of 133,569 bp (1.65%) repeat sequences were identified in the genome of *K. apiculata* 34-9. Among them, the proportion of long terminal repeats (LTRs), long interspersed repeats, short interspersed repeats, and DNA transposon was 0.58, 0.16, 0.01, and 0.36% of the genome, respectively (Supplementary Table 3).

### Global Information of Transcription Profiles

In the present study, we aimed to uncover the mystery of *K. apiculata* responses to cold shock and *P. digitatum* stress, and comparative transcriptomics studies were conducted to gain more insights into the underlying molecular basis. A total of 304.08 Gb clean data were generated from the sequenced 39 yeast samples (at least 21,559,868 reads pairs of each sample), the Q30 was more than 92.54 and 80.54–85.50% of the reads were uniquely mapped to the genome of *K. apiculata* 34-9 (Supplementary Table 4). Since the trials were performed within different time courses, yeast samples obtained at the initial time (incubation for 1 day at 25°C) were marked as 0 h, and their transcriptome data were filtered out, then samples from the rest of the four time periods were conducted for transcription profiles analyses.

**FIGURE 2:**
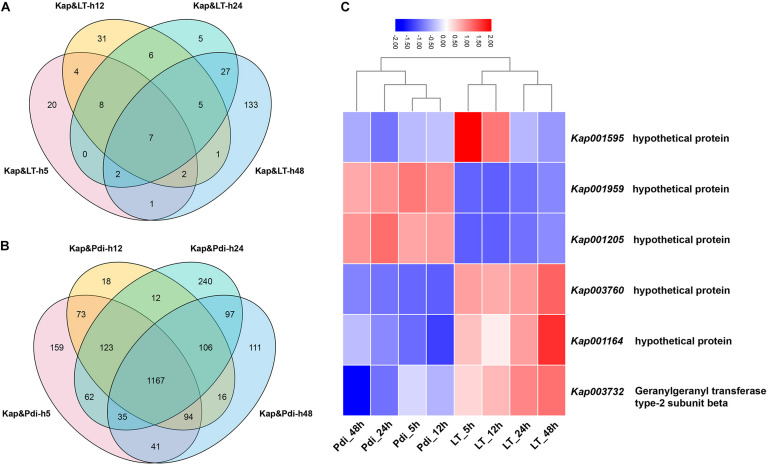
Veen diagram of differentially expressed genes. **(A)** Differentially expressed genes (DEGs) after low temperature treatment. **(B)** DEGs after co-incubation with *Penicillium digitatum.* **(C)** Heat map of co-DEGs (CEGs) between LT and Pdi at the four time points. “Kap,” “LT,” and “Pdi” are *K. apiculata* 34-9 incubated in 25°C, 4°C and co-incubation with *P. digitatum* in 25°C, respectively. “h5,” “h12,” “h24,” and “h48” are yeast samples harvested after incubation for 5, 12, 24, and 48 h, respectively.

The detailed number of DEGs are illustrated in Table 2, and as many as 254 (184 up-regulated, 70 down-regulated) and 2,364 (1,247 up-regulated, 1,117 down-regulated) DEGs were detected in LT and Pdi, respectively. As it can be seen in Table 3, the largest proportion of genes in LT are annotated in cellular component organization or biogenesis, and the maximal percentage of genes in Pdi are annotated in catalytic activity. The majority of DEGs were up-regulated in both treatments (Figures 2A,B), with nearly 72.44 and 52.75% percentage of all the DEGs, respectively. In addition, the largest number of DEGs in LT was found at 48 h, and Pdi was at 24 h. Genes differentially expressed at two or more time points were defined as CEGs (co-differentially expressed genes), a total of six CEGs (three up-regulated genes and three down-regulated genes) appeared in both LT and Pdi at the four time points (Figure 2C), and five CEGs were annotated as encoding hypothetical protein, with only one gene (*Kap003732*) annotated as encoding geranylgeranyl transferase type-2 subunit beta. Here, we found the three up-regulated genes and the three down-regulated genes might be involved in biotic and abiotic stresses responses of *K. apiculata*.

**TABLE 2:** Number of differentially expressed genes of RNA-seq samples.

**Comparisons**	**Upregulated DEGs**	**Downregulated DEGs**	**Total DEGs**
Kap&LT-h5	11	33	44
Kap&LT-h12	40	24	64
Kap&LT-h24	43	17	60
Kap&LT-h48	147	31	178
Kap&Pdi-h5	989	765	1,754
Kap&Pdi-h12	893	716	1,609
Kap&Pdi-h24	951	891	1,842
Kap&Pdi-h48	905	762	1,667

*“Kap,” K. apiculata 34-9 incubated in 25°C. “LT,” K. apiculata 34-9 incubated in 4°C (low temperature treatment). “Pdi,” K. apiculata 34-9 incubated with P. digitatum in 25°C (co-incubation with P. digitatum treatment), co-. “h5,” “h12” “h24,” “h48” are yeast samples harvested after incubation for 5, 12, 24, and 48 h, respectively.*

**TABLE 3:** Number and proportion of genes in each GO category between LT and Pdi.

**GO category**	**GO term**	**LT**		**Pdi**	
		**Gene number**	**Percentage (%)**	**Gene number**	**Percentage (%)**
Cellular component	Endomembrane system	13	5.2	236	10.6
	Organelle lumen	62	24.6	338	15.1
	Non-membrane-bounded organelle	76	30.2	424	19.0
	Ribonucleoprotein complex	52	20.6	224	10.0
	Membrane-enclosed lumen	62	24.6	338	15.1
Molecular function	Catalytic activity	64	25.4	717	32.1
	Oxidoreductase activity	6	2.4	121	5.4
	Catalytic activity, acting on RNA	17	6.7	88	3.9
Biological process	Protein-containing complex localization	10	4.0	45	2.0
	Biosynthetic process	43	17.1	571	25.6
	Small molecule metabolic process	15	6.0	265	11.9
	Oxidation-reduction process	6	2.4	136	6.1
	Cellular component organization or biogenesis	92	36.5	615	27.5
	Cellular component biogenesis	70	27.8	368	16.5

**FIGURE 3:**
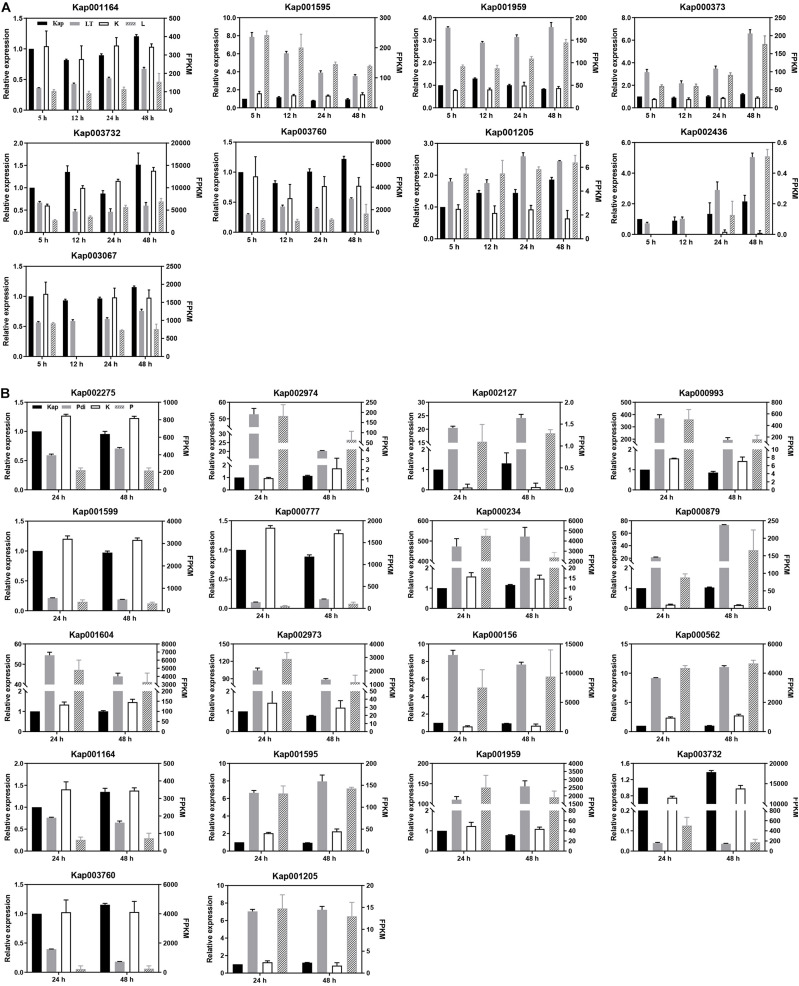
qRT-PCR analyses of DEGs. **(A)** Low temperature treatment. **(B)** Co-incubation with *P. digitatum* treatment. “Kap,” “LT,” and “Pdi” are the qRT-PCR results (left *Y*-axis) of differentially expressed genes, respectively. “K,” “L,” and “P” are the transcription expression abundance (FPKM, right *Y*-axis) of the differentially expressed genes in the RNA-seq results, respectively.

Even though the counts of DEGs generated from qRT-PCR were not the same as FPKMs generated from RNA-seq, they nearly all showed the same trends (Figure 3). The high correlation between qRT-PCR and RNA-seq data were a validation of the generated RNA-seq results.

### Functional Category Analyses of Differentially Expressed Genes Responded to Low Temperature

**FIGURE 4:**
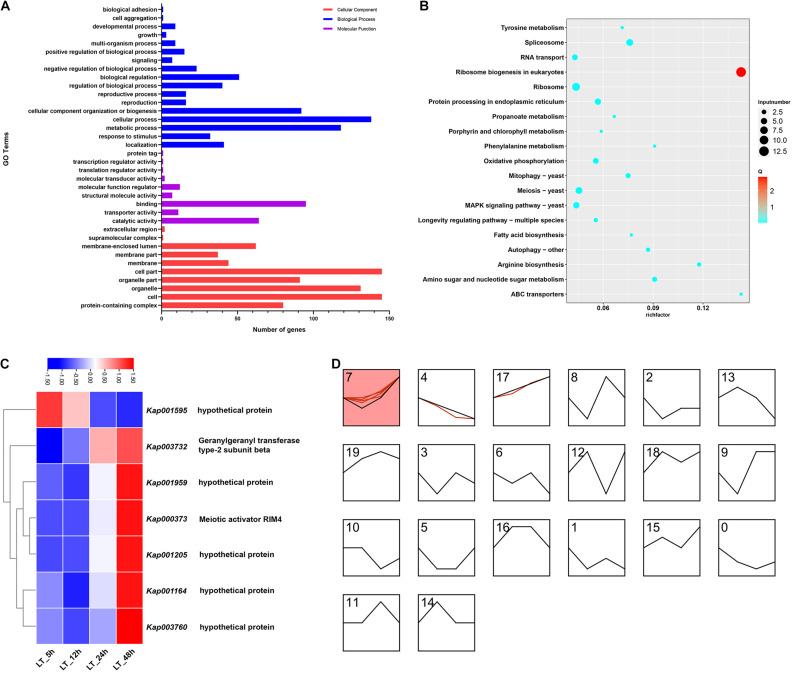
Bioinformatics analyses of DEGs of *K. apiculata* with low temperature treatment. **(A)** GO category of total DEGs. **(B)** KEGG enrichment pathway of total DEGs. **(C)** Heat map of common DEGs. **(D)** Expression patterns analysis of DEGs.

A total of 451 GO terms were obtained in the GO functional category analyses, the number of GO terms of biological process, cellular component and molecular function were 159, 161, and 131, respectively (Figure 4A). For biological process annotation, cellular process (GO: 0009987) and metabolic process (GO: 0008152) were the most plentiful terms, and terms annotated as response to stimulus (GO: 0050896) were also abundant. Meanwhile, binding (GO: 0005488), catalytic activity (GO: 0003824), and molecular function regulator (GO: 0098772) were largely associated terms in the annotation of molecular function. Moreover, cell part (GO: 0044464), protein-containing complex (GO: 0032991), and membrane-enclosed lumen (GO: 0031974) were the most involved terms for cellular component.

For KEGG enrichment analyses, ribosome biogenesis in eukaryotes (KEGG: sce03008), ABC transporters (KEGG: sce02010), and arginine biosynthesis (KEGG: sce00220) were the significantly enriched pathways, they all showed a higher enrichment factor (Figure 4B). In addition, phenylalanine metabolism (KEGG: sce00360) pathway was also enriched in the analyses.

There were 7 CEGs in the four time points in LT (Figure 2A), and the normalized expression counts of the 7 CEGs were visualized in a heatmap, and the expression patterns were clustered in STEM (Figures 4C,D), the 7 CEGs were grouped in 3 clusters (7, 4, 17), gene *Kap003732* remained up-regulated throughout the entire treatments. Interestingly, we also observed one CEG (*Kap003067*) with annotation as heat shock protein 78 (HSP78) was downregulated at 5, 12, and 48 h, but it was not detected differentially expressed at 24 h.

### Functional Category Analyses of Differentially Expressed Genes Responded to *P. digitatum*

**FIGURE 5:**
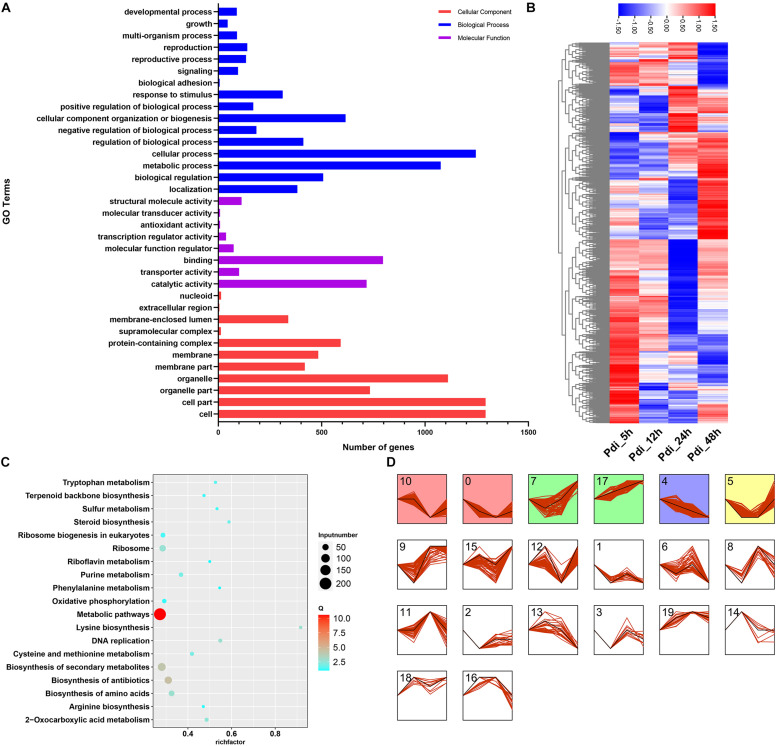
Bioinformatics analyses of DEGs of *K. apiculata* after co-incubation with *P. digitatum*. **(A)** GO category of total DEGs. **(B)** KEGG enrichment pathway of total DEGs. **(C)** Heat map of common DEGs. **(D)** Expression patterns analysis of DEGs.

**FIGURE 6:**
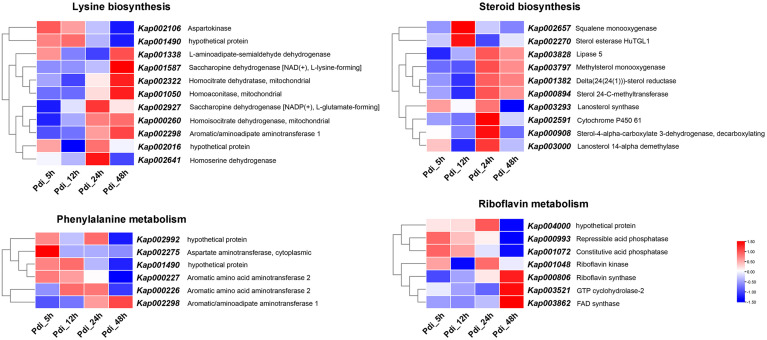
The expression of lysine biosynthesis, phenylalanine metabolism, riboflavin metabolism, and steroid biosynthesis of *K. apiculata* co-incubation with *P. digitatum* treatment.

Because there were many DEGs at different time points, the CEGs from Pdi were utilized for GO category to investigate the possible functions analyses. Results from GO category analyses were on a same occasion to LT, the CEGs were primarily annotated in biological process and cell components (Figure 5A). To get a better understanding of the CEGs, the entire 1,167 CEGs at the four time points in Pdi were employed for further analyses. The normalized expression counts of the 1,167 CEGs were visualized in a heatmap and expression patterns were clustered in STEM (Figures 5B,D), and the 1,167 CEGs were clustered into 20 different groups, group 17 (79 CEGs) was observed up-regulated throughout the entire treatments (Figure 5D). We also performed KEGG enrichment analyses to detect the potential biological pathways, and the results showed that many DEGs were annotated to metabolic pathways (KEGG: sce01100) and biosynthesis of secondary metabolites (sce01110) (Figure 5C). Moreover, lysine biosynthesis (KEGG: sce00300) pathway was enriched with the highest factor, and steroid biosynthesis (KEGG: sce00100) followed. CEGs involved in lysine biosynthesis (KEGG: sce00300), steroid biosynthesis (KEGG: sce00100), phenylalanine metabolism (KEGG: sce00360), and riboflavin metabolism (KEGG: sce00740) KEGG pathways were selected and the FPKM were normalized and visualized in a heatmap (Figure 6).

We also performed GO category and KEGG enrichment analyses of each individual time point. DEGs were associated with catalytic activity (GO: 0003824), hydrolase activity (GO: 0016787), and phosphatase activity (GO: 0016791) after co-incubation for 5, 12, and 24 h (Supplementary Figures 1–3). In addition, DEGs were involved in co-translational protein targeting to membrane (GO: 0006613), sodium ion transport (GO: 0006814), and others after co-incubation for 48 h (Supplementary Figure 4).

### Comparison of Gene Expression Analyses

In the present study, two different kinds of treatments (or stresses) were carried out on *K. apiculata* yeast, and the expression levels were obtained by RNA-seq. A comparison was conducted using WEGO to extend a comprehensive understanding of these treatments, and the detailed GO terms were shown in Table 2. The number of DEGs annotated to catalytic activity (GO: 0003824) of LT and Pdi were 64 (25.4%) and 717 (32.1%), respectively. However, a higher percentage of DEGs annotated to catalytic activity (acting on RNA) (GO: 0140097) of LT than Pdi, with proportion of 6.6 and 3.9%, respectively. DEGs annotated to oxidoreductase activity (GO: 0016491) of LT and Pdi were 6 (2.4%) and 121 (5.4%), respectively. The percentage of DEGs annotated to the cellular component term of LT were mainly reduced compared to Pdi, excluding the endomembrane system.

## Discussion

Here, we present a chromosome level genome with combination of next-generation sequencing (previous data) and Hi-C sequencing to address the limited information of *K. apiculata* 34-9 genome. The newly re-assembled chromosome level genome of *K. apiculata* 34-9 exhibited appreciable improvements in completeness compared to the previous assembled draft genome (Chen et al., 2017), with 228 number of genes added, and more proportion of repeat sequences (1.65%) were annotated. The freshly annotated genes might be owing to the improvements of the continuity of the draft genome, and more useful sequences were obtained. Moreover, it was also because of the development of the algorithm of the software and the updated genome annotation database (Grünberger et al., 2019; Liu T. et al., 2021). The new genome was assembled in scaffolds N50 and contig N50 of 1,349,021 and 104,210 bp, respectively, which were more meticulously examined than those of the former version (scaffolds N50 and contig N50 were 279,578 and 140,302 bp, respectively). In addition, the gene number was near the average number of genes of available sequenced *Hanseniaspora* species (about 4,223 genes) inferred in a previous study (Guaragnella et al., 2020).

RNA-seq had been widely used to study the transcriptome profiles during different treatments (Fracasso et al., 2016; Deng et al., 2018). Many studies were usually performed to detect the transcriptome changes of pythopathogens in response to the presence of biocontrol agents (Gkarmiri et al., 2015; Liu D. et al., 2019). However, few studies have focused on the transcriptome of the biocontrol agents (especially yeast) response to phytopathogens, thus it posed a mystery of these organisms. Transcriptome profiling of *Chaetomium golbosum* in the presence of *Bipolaris sorokiniana* demonstrated that genes related to hydrolytic activity and some secondary metabolites might play a role in antagonistic mechanisms in the antagonist-pathogen interaction (Darshan et al., 2020). In this study, transcriptional profiles of *K. apiculata* co-incubation with *P. digitatum* under different time points were also performed to evaluate the relevant molecular responses; a total of 2,364 genes were found to be differentially expressed in the presence of *P. digitatum*. After co-incubation with *P. digitatum* at 5, 12, 24, and 48 h, a number of 989, 893, 951, and 905 DEGs were up-regulated and 765, 716, 891, and 762 DEGs were down-regulated, respectively. The temporal transcriptome analyses detected numerous candidate genes associated with the response to *P. digitatum* stress. Gene *Kap002298* annotated as aromatic/aminoadipate aminotransferase 1 was involved in lysine biosynthesis and phenylalanine metabolism, and it remained up-regulated with the extension of co-incubation time. Phenylalanine metabolism was important for organisms to survive the stresses (Tonnessen et al., 2015; Solekha et al., 2020). Our results revealed that the significant changes of DEGs associated with vital metabolic pathways such as steroid biosynthesis and riboflavin metabolism among different time points, indicating that it must be a complex regulatory mechanism of *K. apiculata* response to *P. digitatum* stress. The transcriptome data present here might work as a valuable molecular property for future studies.

The inhibitory effect of antimicrobial agents is usually affected by environmental circumstances (Liu J. et al., 2013). In addition, transcriptome analysis has been employed to study the responses of biocontrol agents to abiotic stresses (Spadaro and Droby, 2016; Dai et al., 2021). In this study, transcriptional analyses of biocontrol yeast *K. apiculata* 34-9 between different temperatures (4° and 25°C) were also conducted, and approximately 6.33% of the total genes with 254 DEGs were found after LT treatment at four different time points. Previous studies have shown that ABC transporters play vital roles in diverse biological processes, such as mitochondrial iron homoeostasis, fatty acid metabolism, and mRNA translation (Klein et al., 2011; Do et al., 2018). In the present study, we found that ABC transporters were significantly enriched in the KEGG analyses, they might be involved in the adaption of *K. apiculata* to low temperatures. Heat shock proteins (HSPs) are essential for many cellular processes and organisms to survive stress conditions (Mittal et al., 2011; Park and Seo, 2015). Von Janowsky et al. (2006) responded that HSP78 has a protective role and it could cooperate with the molecular chaperone Ssc1 for disaggregation activity of *S. cerevisiae* during heat stress. In our transcriptome analyses, a protein homologous to HSP78 was down-regulated after being exposed in LT treatment for 5, 12, and 48 h, respectively. It could be inferred that HSP78 of *K. apiculata* might function in the negative regulation of *K. apiculata* to LT stress. Different transcriptomic studies have shown that several genes were considered as cold stress markers, including *NSL1* and *HSP12*, which were up-regulated after cold shock (Tai et al., 2007; Tronchoni et al., 2014). In this study, a maintained up-regulated gene (*Kap003732*) and a maintained down-regulated gene (*Kap001595*) were detected in LT responses, however, these two genes were not significantly enriched in either pathway exhibited in this part. It is possible that the expression changes of these two genes at different time points could be indicators of *K. apiculata* response to LT stress in a short-term adaptation. Low temperature-responsive transcriptome analyses in this study may provide a data-based model for elucidating the molecular mechanisms of *K. apiculata* low temperature tolerance.

## Conclusion

In conclusion, a chromosome resolution genome of *K. apiculata* was obtained, and transcriptome expression profiles of *K. apiculata* 34-9 under low temperature and co-incubation with *P. digitatum* were obtained. These results may establish a foundation for the genome study of *Hanseniaspora* spp., and the regulatory mechanisms of low temperature adaptation. Meanwhile, the transcriptome data of *K. apiculata* co-incubation with *P. digitatum* may extend our knowledge on *Kloeckera* yeast as biocontrol agents against citrus green mold, and the involved mechanisms might be a study model on other phytopathogens. These findings might provide relevant information of biocontrol agents and postharvest citrus storage and preservation.

## Data Availability Statement

The datasets presented in this study can be found in online repositories. The names of the repository/repositories and accession number(s) can be found below: BioProjects accession numbers PRJNA667993 and PRJNA668195.

## Author Contributions

CL: conceptualization, writing—review and editing, supervision, and funding acquisition. ZT: conceptualization, methodology, data curation, and writing—original draft. YD, FY, JZ, SL, and DZ: methodology, validation, investigation, and data curation. All authors contributed to the article and approved the submitted version.

## Conflict of Interest

The authors declare that the research was conducted in the absence of any commercial or financial relationships that could be construed as a potential conflict of interest.

## Publisher’s Note

All claims expressed in this article are solely those of the authors and do not necessarily represent those of their affiliated organizations, or those of the publisher, the editors and the reviewers. Any product that may be evaluated in this article, or claim that may be made by its manufacturer, is not guaranteed or endorsed by the publisher.

## Footnotes

1
http://www.repeatmasker.org

2
http://www.girinst.org/repbase

3
http://rfam.xfam.org/

4
https://www.ncbi.nlm.nih.gov/tools/primer-blast

5
http://www.ncbi.nlm.nih.gov/sra
