# Supplementary material for: Chromosome Genome Sequencing and Comparative Transcriptome-Based Analyses of Kloeckera apiculata 34-9 Unveil the Potential Biocontrol Mechanisms Against Citrus Green Mold
Source: Front Microbiol. 2021 Nov 9;12:752529. doi: 10.3389/fmicb.2021.752529 (PMC8631199; doi:10.3389/fmicb.2021.752529)
Supplement: Supplementary file 1 [file Data_Sheet_1.docx]

**SupplementaryFigures and Tables**

**Supplementary Figures**

**FigureS1. GO category and KEGG enrichment of differentially expressed genes of co-incubation with *P. digitatum* for 5 h**

**FigureS2. GO category and KEGG enrichment of differentially expressed genes of co-incubation with *P. digitatum* for 12 h**

**FigureS3. GO category and KEGG enrichment of differentially expressed genes of co-incubation with *P. digitatum* for 24 h**

**FigureS4. GO category and KEGG enrichment of differentially expressed genes of co-incubation with *P. digitatum* for 48 h**


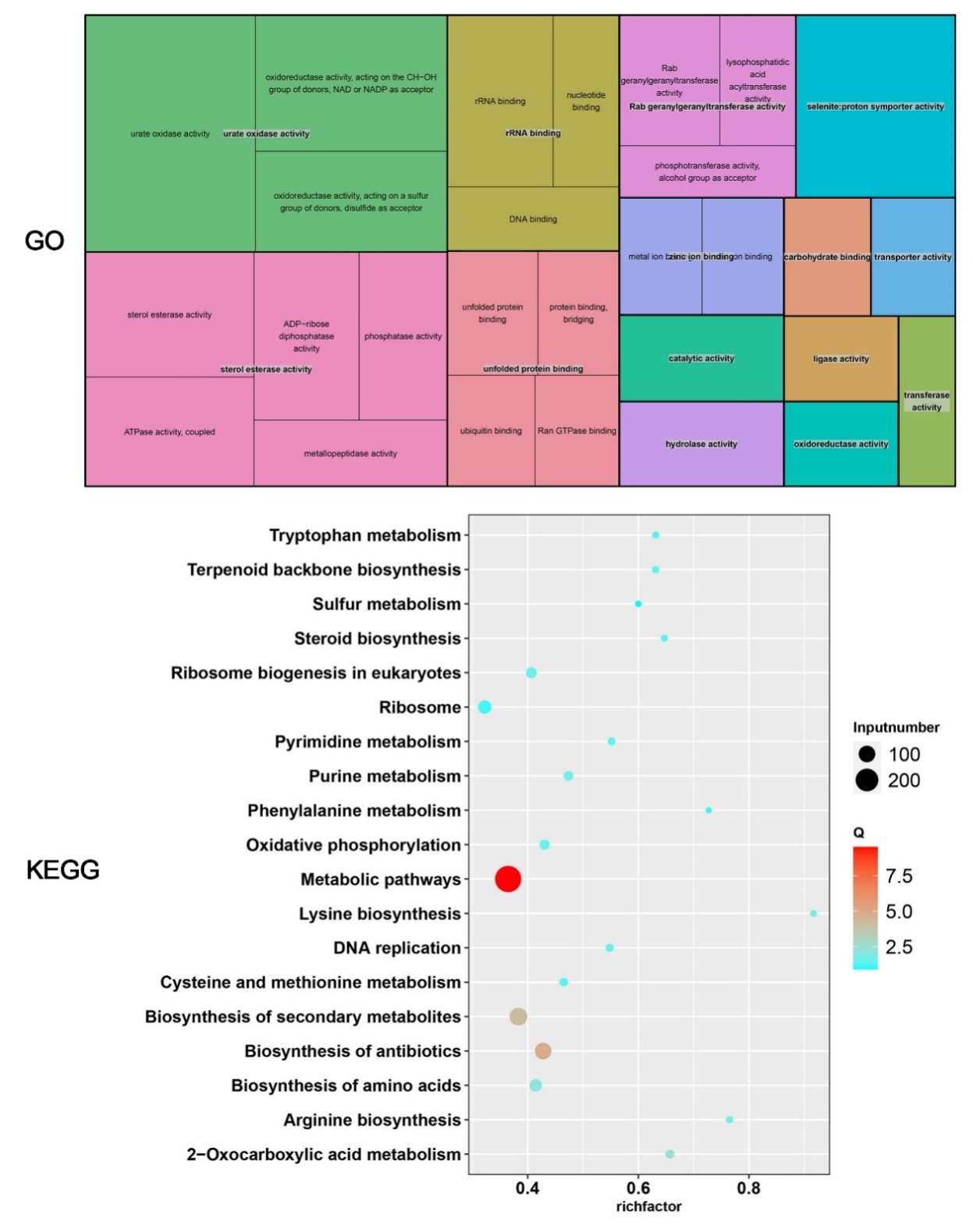


**Figure S1. GO category and KEGG enrichment of differentially expressed genes of co-incubation with *P. digitatum* for 5 h**


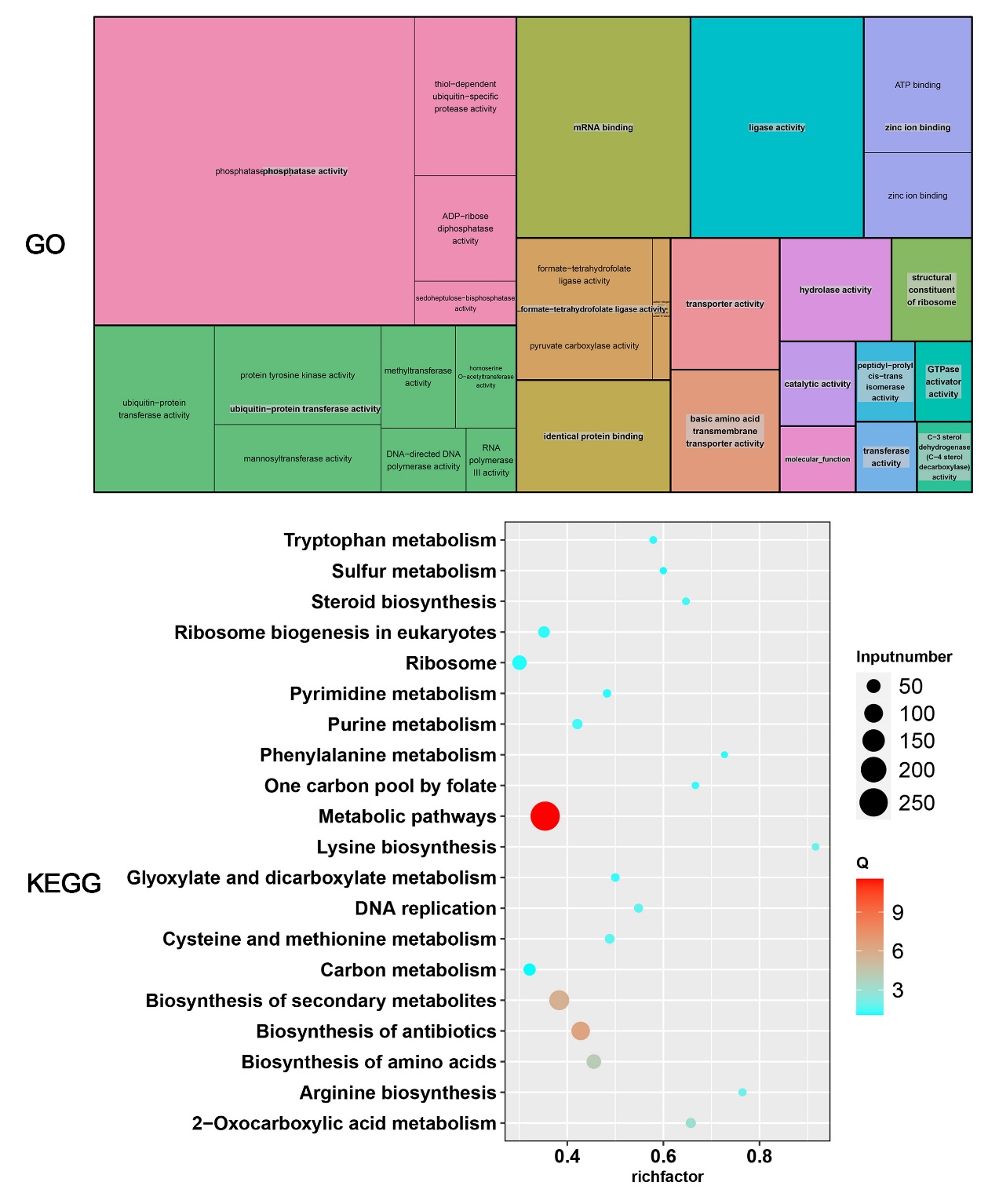


**Fig.S2. GO category and KEGG enrichment of differentially expressed genes of co-incubation with *P. digitatum* for 12 h**


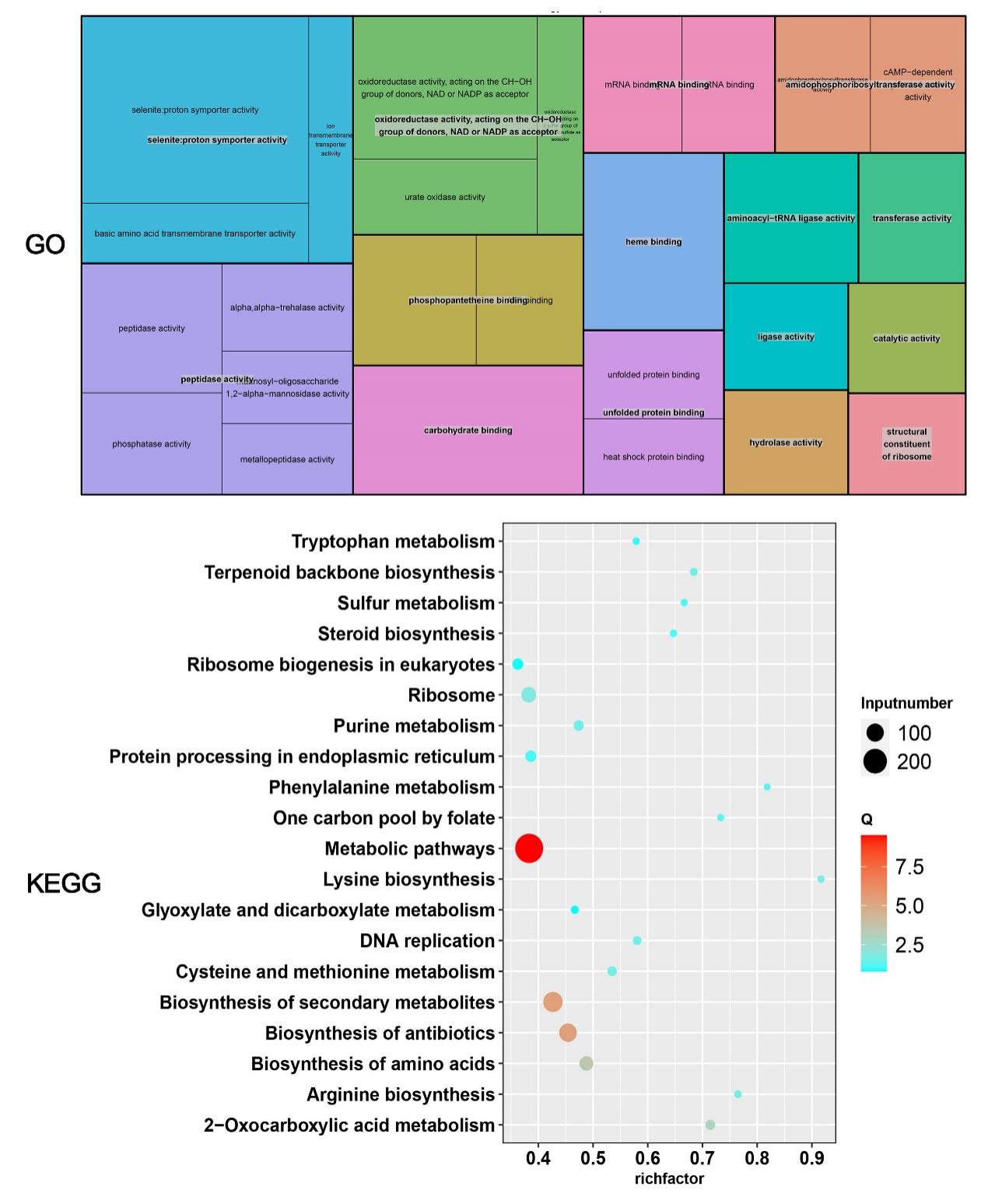


**Fig. S4. GO category and KEGG enrichment of differentially expressed genes of co-incubation with *P. digitatum* for 24 h**


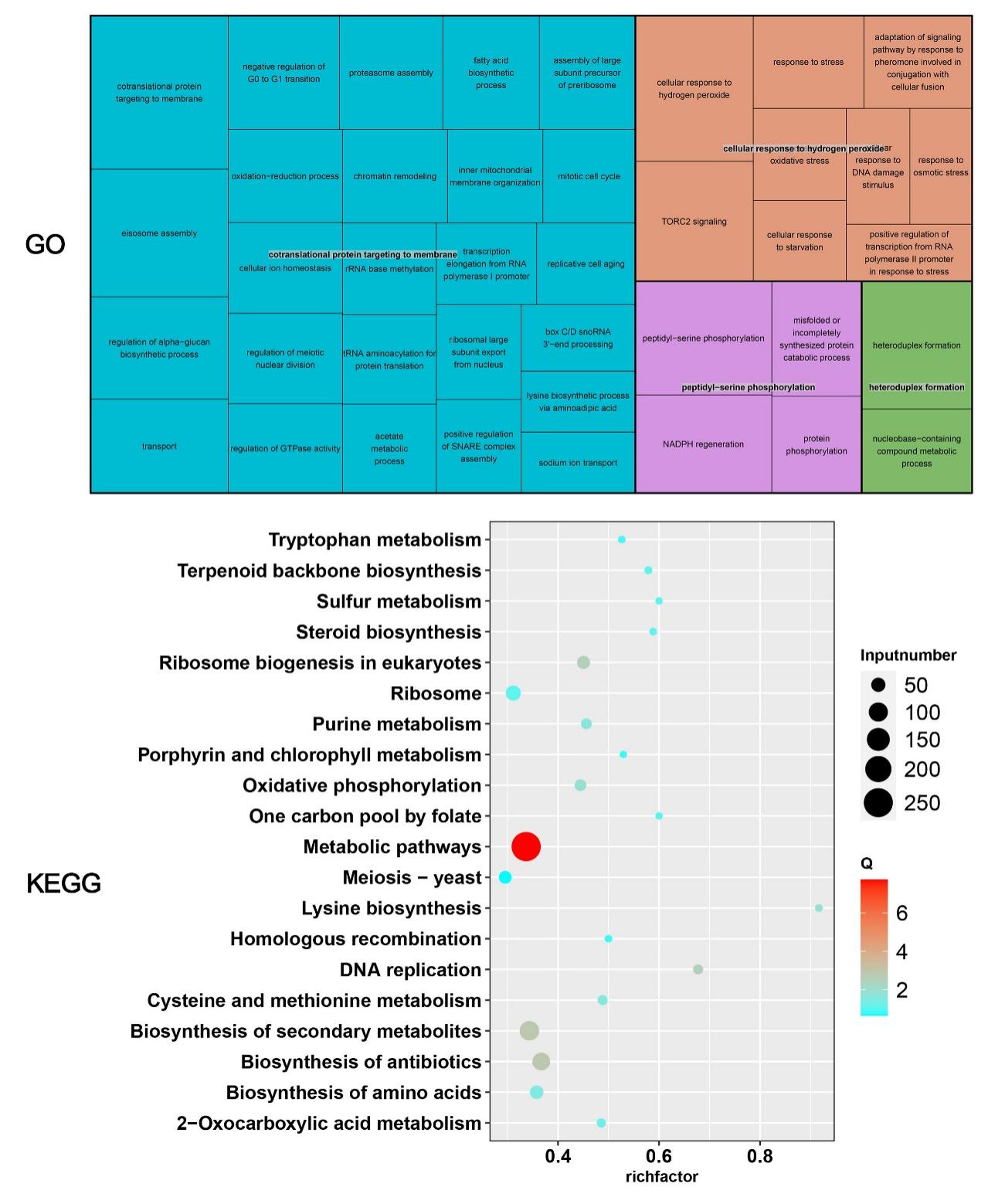


**Fig. S5. GO category and KEGG enrichment of differentially expressed genes of co-incubation with *P. digitatum* for 48 h**

**Supplementary Tables**

**TableS1. Primers used for qRT-PCR reaction**

**Table S2.Assembly of Hi-C sequencing data**

**Table S3. Information of repeat sequences**

**Table S4. Results of RNA sequencing data of *K. apiculata* 34-9**

**TableS1. Primers used for qRT-PCR reaction**

| Primer Name | Sequence (5’ to 3’) | Possible functions/annotation |
| --- | --- | --- |
| Kap1164-F | TCGGCACCAATACCAAATC | hypothetical protein |
| Kap1164-R | CTTGTGGAATGCCAACCT |  |
| Kap1595-F | TGGTTTGCCGTTGTTCTC | hypothetical protein |
| Kap1595-R | TGGAATTGTGCAGGTATCTTT |  |
| Kap1959-F | GACTCCTCTGCTTCACAATC | hypothetical protein |
| Kap1959-R | GATCTGGCAGTGGCAATAG |  |
| Kap0373-F | CCCAAACAGCTCCGTATAAT | Meiotic activator RIM4 |
| Kap0373-R | AGAGGCACTTTGATGGATTC |  |
| Kap3732-F | CGATGGTGGATTTGGTCTT | Geranylgeranyl transferase type-2 subunit beta |
| Kap3732-R | TTTCACTGAGCCACCATTT |  |
| Kap3760-F | CAAGCCTCGGCTCATTTAT | hypothetical protein |
| Kap3760-R | CGCTAGCAGATTGGTATGG |  |
| Kap1205-F | GTGCTGGTCTTGATGATGT | hypothetical protein |
| Kap1205-R | GAGCGAAGAGTCAGATCTATTT |  |
| Kap2436-F | CTAAGCCTGGGTGTAAAGAC | hypothetical protein |
| Kap2436-R | TCGATGCTCTATGGCTACT |  |
| Kap3067-F | CGACAGCATAGGGTTGTTAC | Heat shock protein 78, mitochondrial |
| Kap3067-R | GGCTTCAATGGCTTCATCT |  |
| Kap2275-F | GACACCAGACCACACAAAG | Aspartate aminotransferase, cytoplasmic |
| Kap2275-R | AGGGCGTCTAAACCAGATA |  |
| Kap2974-F | CCGTCACTGCTGATCTATTT | hypothetical protein |
| Kap2974-R | AGATTCAGTGCCTGTTGTAG |  |
| Kap2127-F | GGTGCAGTGGACAAAGAT | hypothetical protein |
| Kap2127-R | CTAGCATTGACTGAGGATATGG |  |
| Kap0993-F | GAAACGACTTCACTCCTATGG | Repressible acid phosphatase |
| Kap0993-R | CAGCTCTGCTGATGAAGTTAT |  |
| Kap1599-F | CATGTCCTGAAGCCCTATTG | Citrate synthase, mitochondrial |
| Kap1599-R | GGGTGTAAGTCCTTTGGTAAG |  |
| Kap0777-F | AGACTCTGAAGACGAAGGT | hypothetical protein |
| Kap0777-R | CTTCCTTGATGACCCAAGAG |  |
| Kap0234-F | ATTGCTGGTGGTTGGTTAG | Ammonium transporter MEP2 |
| Kap0234-R | GCAGCATCAGTACCCATTT |  |
| Kap0879-F | TGGGTTCCTTGCAGAATATG | Flocculation protein FLO5 |
| Kap0879-R | CACTTCCTTGCCCTGAATAC |  |
| Kap1604-F | AAGTTGGTCCATGTGAAGG | Glutamine synthetase |
| Kap1604-R | GTTCTTGGTGGAGACGTTAG |  |
| Kap2973-F | CTTCTTTCTCTGGCTCTTCTT | hypothetical protein |
| Kap2973-R | GCTTCGGTGGAGGATTTAG |  |
| Kap0156-F | GGGTTTCGCTATTTCCTTCT | Hexose transporter 2 |
| Kap0156-R | ACCACATTTCGTTGACTTCT |  |
| Kap0562-F | CTTTAGAAGCTGCCCGTATT | 60S ribosomal protein L10 |
| Kap0562-R | CAAGCACCTCTCATACCTTG |  |
| actKap3452-F | GAGCTCCAGAAGCCTTATTC | actin-domain-containing protein |
| actKap3452-R | GGCAATACCTGGGAACATAG |  |

**Table S2.Assembly of Hi-C sequencing data**

| Group | Sequence Number | Sequence Length (bp) |
| --- | --- | --- |
| Lachesis Group 1 | 9 | 2,281,138 |
| Lachesis Group 2 | 1 | 1,362,551 |
| Lachesis Group 3 | 3 | 738,699 |
| Lachesis Group 4 | 5 | 1,138,124 |
| Lachesis Group 5 | 7 | 1,073,375 |
| Lachesis Group 6 | 1 | 904,535 |
| Lachesis Group 7 | 3 | 565,349 |
| Total Sequences Clustered (Ratio %) | 29 (64.44) | 8,063,771 (99.51%) |
| Total Sequences Ordered and Oriented (Ratio %) | 29 (100) | 8,063,771 (100%) |

**Table S3. Information of repeat sequences**

| Type | Repeat size (bp) | % of genome |
| --- | --- | --- |
| Trf | 44,676 | 0.55 |
| RepeatMasker | 52,610 | 0.65 |
| RepeatProteinMask | 43,140 | 0.53 |
| De novo | 0 | 0 |
| Total | 133,569 | 1.65 |

**Table S4. Results of RNA sequencing data of *K. apiculata* 34-9**

| Sample | Clean Reads Pairs | Mapped reads (%) | Clean base (bp) | Length | Q30 (%) | GC (%) |
| --- | --- | --- | --- | --- | --- | --- |
| 0h-1 | 25,259,780 | 83.20 | 7,556,581,662 | 149 | 93.8 | 36.5 |
| 0h-2 | 25,101,936 | 83.23 | 7,513,966,060 | 149 | 93.7 | 36.75 |
| 0h-3 | 38,761,563 | 84.63 | 11,516,993,152 | 148 | 93.3 | 36.5 |
| LT5h-1 | 28,339,328 | 83.26 | 8,477,922,934 | 149 | 93.85 | 36.75 |
| LT5h-2 | 24,767,845 | 84.00 | 7,414,856,176 | 149 | 93.95 | 36.85 |
| LT5h-3 | 24,929,355 | 83.57 | 7,466,527,312 | 149 | 93.9 | 37.35 |
| Kap5h-1 | 24,395,115 | 84.32 | 7,295,163,622 | 149 | 93.95 | 37.3 |
| Kap5h-2 | 27,296,386 | 84.23 | 8,172,443,842 | 149 | 93.95 | 36.75 |
| Kap5h-3 | 26,899,062 | 83.70 | 8,055,506,734 | 149 | 93.95 | 36.9 |
| Pdi5h-1 | 27,337,119 | 83.89 | 8,177,707,184 | 149 | 93.75 | 38.2 |
| Pdi5h-2 | 28,031,153 | 82.23 | 8,390,678,096 | 149 | 93.7 | 37.75 |
| Pdi5h-3 | 26,891,935 | 83.88 | 8,049,403,458 | 149 | 93.75 | 37.75 |
| LT12h-1 | 28,736,501 | 83.92 | 8,600,504,462 | 149 | 94.1 | 36.95 |
| LT12h-2 | 27,390,723 | 83.67 | 8,198,936,262 | 149 | 94.1 | 37.15 |
| LT12h-3 | 27,890,031 | 83.51 | 8,344,746,264 | 149 | 93.8 | 37.15 |
| Kap12h-1 | 24,673,775 | 84.79 | 7,388,343,668 | 149 | 94.15 | 36.7 |
| Kap12h-2 | 28,317,560 | 83.46 | 8,481,623,350 | 149 | 93.5 | 36.9 |
| Kap12h-3 | 26,226,192 | 85.03 | 7,846,740,932 | 149 | 93.85 | 36.7 |
| Pdi12h-1 | 24,854,459 | 83.47 | 7,441,042,390 | 149 | 92.55 | 37.6 |
| Pdi12h-2 | 26,150,115 | 82.67 | 7,820,531,766 | 149 | 92.85 | 37.55 |
| Pdi12h-3 | 22,135,391 | 83.52 | 6,625,133,060 | 149 | 93 | 37.65 |
| LT24h-1 | 23,776,244 | 84.26 | 7,112,727,226 | 149 | 93.15 | 37.05 |
| LT24h-2 | 29,776,013 | 84.00 | 8,906,533,496 | 149 | 93.15 | 36.7 |
| LT24h-3 | 23,884,287 | 84.20 | 7,147,518,066 | 149 | 92.9 | 36.65 |
| Kap24h-1 | 26,571,953 | 84.59 | 7,950,897,442 | 149 | 93.15 | 36.65 |
| Kap24h-2 | 25,955,323 | 85.04 | 7,766,768,002 | 149 | 93.05 | 36.65 |
| Kap24h-3 | 24,011,550 | 84.74 | 7,178,998,490 | 149 | 93 | 36.65 |
| Pdi24h-1 | 24,412,086 | 80.54 | 7,306,045,068 | 149 | 92.95 | 38.55 |
| Pdi24h-2 | 26,972,726 | 82.23 | 8,077,063,476 | 149 | 92.8 | 38.15 |
| Pdi24h-3 | 27,162,501 | 83.18 | 8,129,496,280 | 149 | 93.2 | 38.15 |
| LT48h-1 | 27,002,749 | 85.07 | 8,063,949,630 | 149 | 93.85 | 36.55 |
| LT48h-2 | 25,878,763 | 85.50 | 7,744,633,598 | 149 | 93.05 | 36.55 |
| LT48h-3 | 28,873,540 | 85.08 | 8,633,177,564 | 149 | 93.05 | 36.8 |
| Kap48h-1 | 24,299,396 | 84.82 | 7,272,635,722 | 149 | 93.1 | 36.55 |
| Kap48h-2 | 25,255,359 | 84.79 | 7,552,893,466 | 149 | 93.2 | 36.55 |
| Kap48h-3 | 22,101,131 | 85.03 | 6,597,348,340 | 149 | 93.05 | 37.05 |
| Pdi48h-1 | 21,559,868 | 80.62 | 6,452,637,882 | 149 | 94.65 | 38.3 |
| Pdi48h-2 | 22,943,724 | 81.46 | 6,870,464,346 | 149 | 94.45 | 37.95 |
| Pdi48h-3 | 21,670,453 | 81.39 | 6,478,108,636 | 149 | 94.95 | 38.25 |
